# In-situ growth of low-dimensional perovskite-based insular nanocrystals for highly efficient light emitting diodes

**DOI:** 10.1038/s41377-023-01112-7

**Published:** 2023-03-03

**Authors:** Hao Wang, Weidong Xu, Qi Wei, Si Peng, Yuequn Shang, Xianyuan Jiang, Danni Yu, Kai Wang, Ruihua Pu, Chenxi Zhao, Zihao Zang, Hansheng Li, Yile Zhang, Ting Pan, Zijian Peng, Xiaoqin Shen, Shengjie Ling, Weimin Liu, Feng Gao, Zhijun Ning

**Affiliations:** 1 School of Physical Science and Technology & Shanghai Key Laboratory of High-resolution Electron Microscopy, ShanghaiTech University, Shanghai, China; 2 Institute of Flexible Electronics, Northwestern Polytechnical University, Xi’an, China; 3 Department of Physics, Chemistry and Biology (IFM), Linköping University, Linköping, Sweden

**Keywords:** Lasers, LEDs and light sources, Displays

## Abstract

Regulation of perovskite growth plays a critical role in the development of high-performance optoelectronic devices. However, judicious control of the grain growth for perovskite light emitting diodes is elusive due to its multiple requirements in terms of morphology, composition, and defect. Herein, we demonstrate a supramolecular dynamic coordination strategy to regulate perovskite crystallization. The combined use of crown ether and sodium trifluoroacetate can coordinate with A site and B site cations in ABX_3_ perovskite, respectively. The formation of supramolecular structure retard perovskite nucleation, while the transformation of supramolecular intermediate structure enables the release of components for slow perovskite growth. This judicious control enables a segmented growth, inducing the growth of insular nanocrystal consist of low-dimensional structure. Light emitting diode based on this perovskite film eventually brings a peak external quantum efficiency up to 23.9%, ranking among the highest efficiency achieved. The homogeneous nano-island structure also enables high-efficiency large area (1 cm^2^) device up to 21.6%, and a record high value of 13.6% for highly semi-transparent ones.

## Introduction

The regulation of perovskite growth significantly prompts the performance enhancement of perovskite-based optoelectronic devices^1–3^. In recent years, benefits from the development of advanced thin-film processing techniques, such as compositional engineering, morphology control, dimensionality manipulation, and surface passivation, the efficiency of perovskite light-emitting diodes develop significantly, and it generates tremendous interest due to its high color purity, high brightness, facile bandgap tunability, and compatibility with low-cost manufacturing technology^4–7^.

Using additives to control crystallization dynamics is an imperative strategy to achieve high-quality film for light-emitting diodes^8–10^. The manipulation of perovskite growth by additives enables the formation of a nano-island structure that facilitates carrier injection and radiative recombination. For low-dimensional structures, the application of additives to regulate perovskite growth enables the elimination of *n* = 1 layered perovskite that radiate slower than those with higher dimensionalities^11,12^. Furthermore, additives utilized for manipulating perovskite growth can also passivate surface defects^13^.

Despite lots of additives being explored to regulate perovskite growth, their function of crystal regulation is still limited. For example, 5-aminovaleric acid is used to form nano-island morphological features based on high-dimensional bulk structure^14^. Additives such as crown ether is added to tune the dimensionality of low-dimensional structure, but it typically shows continuous film morphology^15–18^. Considering the quick carrier injection of nano-island structure and the high luminescent quantum yield of low-dimensional structure, it is desirable to fabricate an insular nanocrystal based on low-dimensional structural emitter that can combine effective carrier injection and high luminescent quantum yield^19–21^. However, the methodology that can precisely regulate the composition and morphology simultaneously remains to be challenging, which can be partially attributed to the fact that most additives can only coordinate with B-site cations^22^. Furthermore, the coordination between additive and cations is rarely studied, and the kinetics of crystallization manipulation by additives is elusive.

In this work, we develop a supramolecular dynamic coordination-induced segmented crystallization for perovskite emitters, in which crown ether and sodium trifluoroacetate are used to coordinate with both A-site and B-site ions of perovskite. In-situ characterization indicates that the coordination retard perovskite nucleation at room temperature and the growth of low-dimensional structure, while at high temperature, the transformation of supramolecular structure enables the release of both A site and B site cations for the secondary step growth. It leads to the growth of insular nanocrystals consist of low-dimensional perovskite, showing both excellent luminescent quantum yield and effective carrier injection. As a result, we are able to achieve high-performance green PeLEDs with peak external quantum efficiency up to 23.9%. In addition, this well-controlled kinetics enables the growth of a large area (1 cm^2^) device showing a high value of 21.6%. This unique nano-island structure attenuates light absorbance, enabling the fabrication of a highly transparent light-emitting diode showing a record high quantum yield up to 13.6%^15,23^.

## Results

### Film structure and morphology

All perovskite thin films are prepared by one-step spin-coating without anti-solvent treatment. The precursor solutions consist of lead (II) bromide (PbBr_2_), cesium bromide (CsBr), FABr, and phenethylammonium bromide (PEABr) in dimethylsulfoxide (DMSO). We select 18-crown-6 (18Cr6) and/or sodium trifluoroacetate (STFA) as the additives. The details for perovskite film deposition are summarized in Supplementary Information (SI). The films prepared by either 18Cr6, STFA, or a combination of the two are denoted as 18Cr6, STFA, and CrTFA, respectively.

**Fig. 1:**
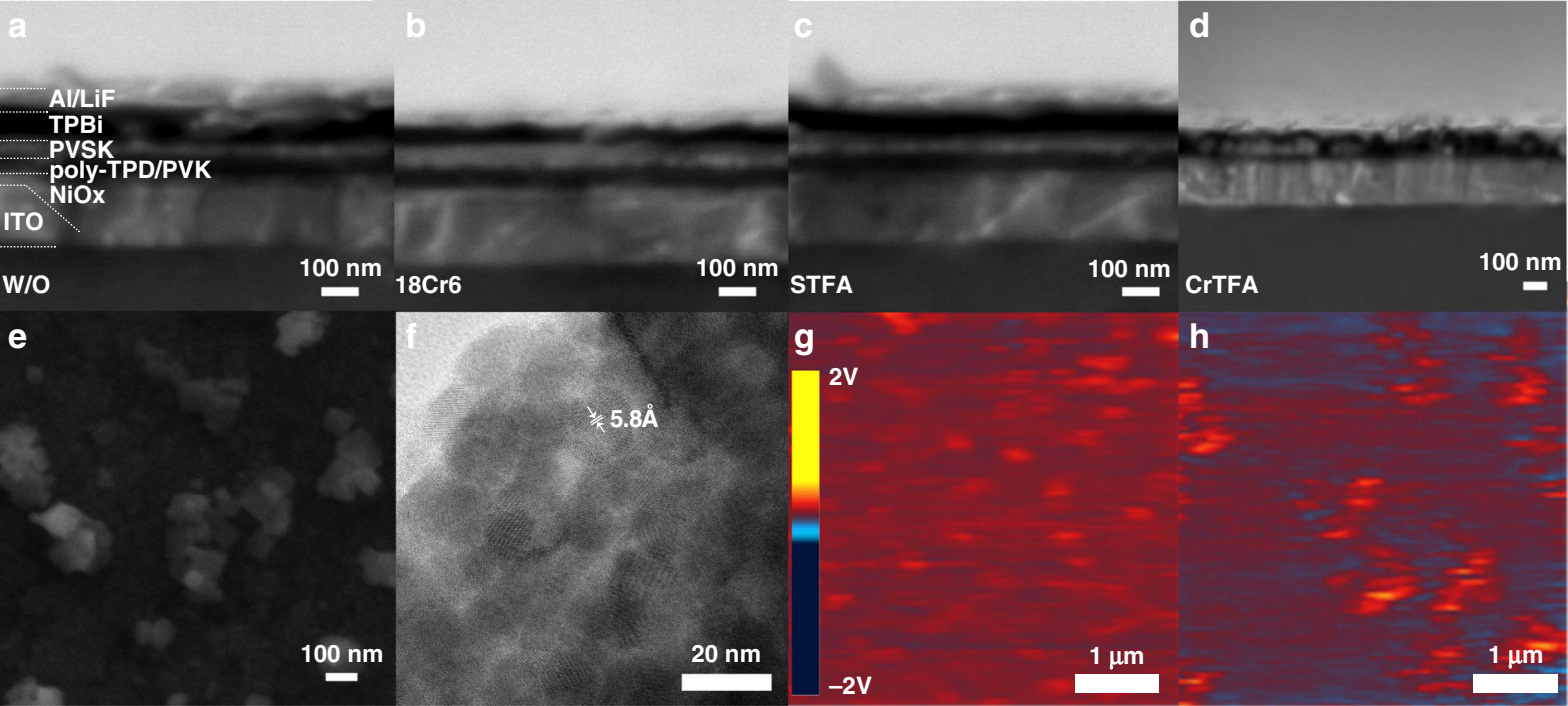
Morphology of perovskite films. Cross-sectional SEM images of devices based on **a** perovskite films without additives, **b** 18Cr6 films, (**c**) STFA films, and **d** CrTFA films. Device configuration: indium tin oxide (ITO;180 nm)/Nickel oxide (NiO_x_; 15 nm)/Poly[N,N’-bis(4-butylphenyl)-N,N’-bis(phenyl)-benzidine] (poly-TPD; 15 nm)/Poly (9-vinylcarbazole) (PVK; 15 nm)/perovskite light emitting layer/1,3,5-Tris (1-phenyl-1H-benzimidazol-2-yl)benzene (TPBi; 40 nm)/Lithium fluoride (LiF; 1.2 nm)/Aluminium (Al; 100 nm). Unlike other films, CrTFA films show discontinuous structure; **e** SEM image of the surface of CrTFA film. **f** TEM of CrTFA films show grain size around 20 nm. AFM-IR (1710 cm^-1^) images of CrTFA films **g** before and **h** after annealing indicate the films becomes discontinuous after annealing

We first characterized the morphology of perovskite films. Figure 1a–d shows scanning electron microscope (SEM) cross-sectional images of all the samples of interest, where the perovskite layers are sandwiched between electron and hole transport layers. The films without additive and with single additive are dense and even, while the CrTFA films show nano-island features with discrete grains (Supplementary Figs. 1 and 2). The discrepancies in film morphology are confirmed by SEM surface image showing well separated square-shaped grains (Fig. 1e).

For deeper insights into the evolution of the local composition of CrTFA films upon annealing, we performed atomic force microscopy-infrared (AFM-IR) spectroscopy. As FA^+^ offers distinct IR signals from the stretching vibration of its C=N double bond (ν(C=N)), we select the corresponding frequency (1710 cm^−1^) as the trace for IR absorption mapping^24^. Before annealing, the CrTFA films show a continuous distribution of FA^+^ with no clear grain boundary, while discrete domains are observable after thermal treatment (Fig. 1g, h), indicating the segmented growth during thermal treatment. In addition, no signal from ν(C=N) is discernable in the dark area of resulting films, indicating the discontinuous film morphology again (Supplementary Fig. 3).

We performed transmission electron microscope (TEM) measurements to evaluate the crystal quality^25,26^. As shown in Fig. 1e, the CrTFA films displays a uniform grain size of ~20 nm. A crystal lattice with an inter-distance of 5.8 Å is clearly observed, corresponding to the (100) plane of CsPbBr_3_ (Fig. 1f)^27^. Fast Fourier Transformation (FFT) shows diffraction spots deriving from the (100) and (200) planes, respectively, indicating high crystallinity of the film (Supplementary Fig. 4a). In contrast, the lattice cannot be clearly seen for the STFA films, indicating poor crystallinity. The 18Cr6 films shows a crystal size of less than 10 nm, much smaller than in the CrTFA films (Supplementary Fig. 4b and 4c). The distinct variation of film morphology indicates the critical role of additives in modifying the crystallization process.

### Film growth tracking

**Fig. 2:**
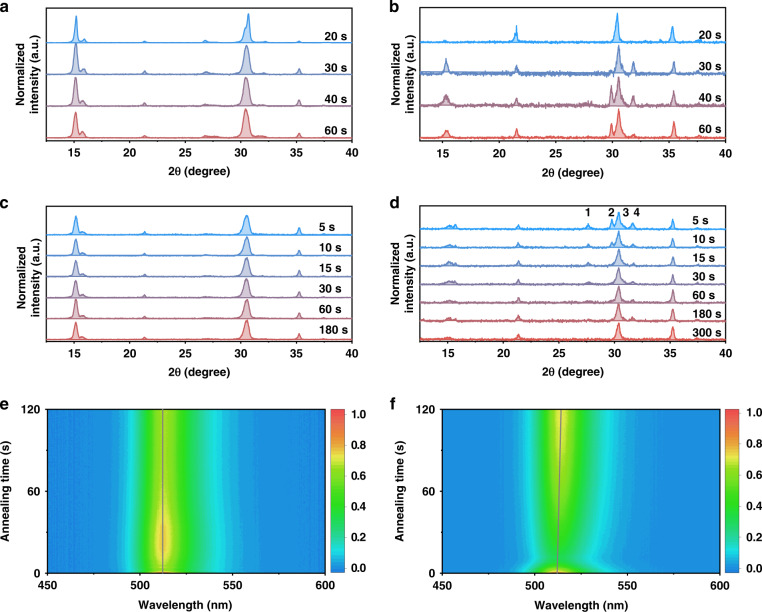
Crystallization process of perovskites. XRD spectra evolution during spin-coating for **a** control and **b** CrTFA films. New peaks from FABr and other components appear for the CrTFA films. XRD spectral evolution during the annealing for **c** control and **d** CrTFA films. These new peaks disappear gradually during the annealing process (Peaks 1, 2, 3, and 4 derive from NaBr, CsBr, PbBr_2_, and FABr, respectively). PL spectral change during annealing for **e** control and **f** CrTFA films. The PL peak shifts to longer wavelength during annealing process of the CrTFA films

To understand the mechanisms underlying the changes in film morphology, we studied the perovskite crystallization dynamics by tracking X-ray diffraction (XRD) evolution as a function of time during spin-coating and annealing (Fig. 2a–d)^28^. The former and latter take 60 s and 300 s, respectively. For all films, the diffraction peaks from the perovskite (200) plane emerge within 20 s and are conserved during the whole annealing process. For the control films, a tiny diffraction peak from FABr is observed after spin coating and quickly disappears once annealing begins. Notably, diffraction peaks of perovskite precursors, including FABr, CsBr, and PbBr_2_, can be detected in the CrTFA films during spin-coating. This is suggestive of an uncompleted reaction and distinct phase separation of the reactants. In the following annealing process, the diffraction peaks from FABr, PbBr_2_, and CsBr gradually disappear over 300 s, indicating a retarded but completed grain growth.

For more insights into the perovskite crystallization during annealing, we perform in-situ PL measurements^29^. Both control and CrTFA films were investigated. As shown in Figs. 2e and 2f, the initial decrease of luminescence intensity during annealing can be ascribed to the increase of non-radiative transition due to lattice vibration^30^. Notably, the emission bands of CrTFA films are gradually red-shift over annealing, in line with the increase of grain size as observed in SEM morphological images^31^. In comparison, the emission peak of control films remains constant. These further confirm the segmented crystallization for CrTFA films.

### Functionalities of additives

To understand the respective roles of 18Cr6 and STFA in determining the crystallization processes of CrTFA films, we further monitor the grain growth in films with single additives during annealing. The results of in-situ XRD measurements are shown in Supplementary Figs. 5 and 6. No diffraction signal from FABr is detectable in the 18Cr6 films, but diffraction is clearly seen in the STFA case even after 300 s annealing. This suggests a phase separation of FABr is due to the interaction between STFA. The control films and the STFA films show a strong diffraction peak from the 2D structure after spin coating, while no peak is observed for the 18Cr6 films.

**Fig. 3:**
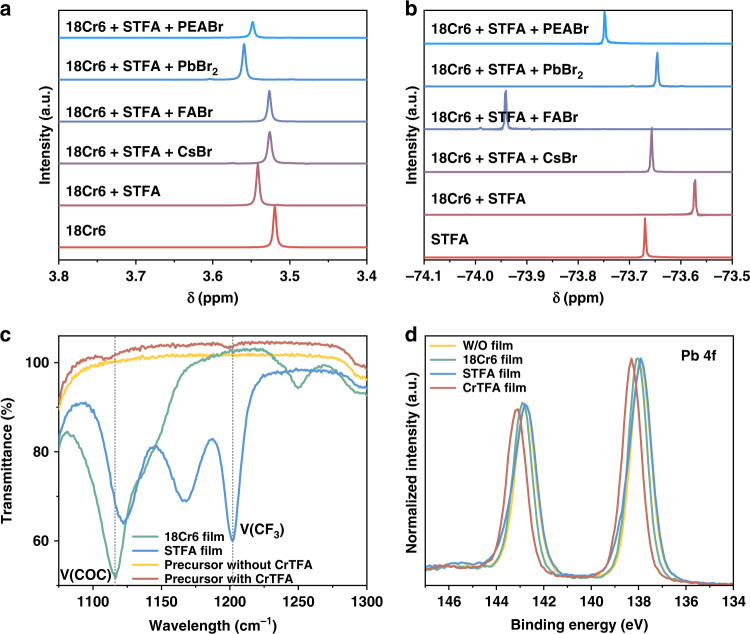
Chemical interaction between additives and perovskite precursors. **a** ^1^H and **b** ^19^F NMR spectra of solutions with STFA or 18Cr6 and a combination of them (CrTFA). Obvious shifts of NMR peaks indicate interactions between the components. **c** IR spectra of an 18Cr6 films, an STFA films, as well as precursors with and without CrTFA. The peak shift indicates interaction between these components. **d** XPS spectra of the thin films. The shift of the Pb 4f peak shows the interaction between lead and the additives

To understand how additives influence crystal growth, we performed Fourier-transform infrared spectroscopy (FT-IR) measurement on the 18Cr6 films, STFA films, and perovskite precursors with and without CrTFA. Compared to the spectrum of the reference sample of additive alone, the stretching vibration ν(C-O-C) of 18Cr6 in perovskite precursor shifts to a higher wavenumber (Fig. 3c), indicating the interaction between the oxygen on 18Cr6 and cations from additives^32^. The peak of the stretching vibration ν(CF_3_) of STFA shifts to smaller wavenumber in the precursor, which may derive from the interaction between carboxyl moieties and cations such as ammonium and metal cations^33^.

We further performed ^1^H and ^19^F nuclear magnetic resonance (NMR) measurements to investigate the interaction between the additives and perovskite precursors. D_6_-DMSO solutions with STFA or 18Cr6 and a combination of them (CrTFA) were prepared for reference. The respective addition of PbBr_2_, FABr, CsBr, or PEABr in the CrTFA solution brings about distinct changes in the chemical shift of -CH_2_- (on 18Cr6) (Fig. 3a). This can be ascribed to the interaction between oxygen atoms and cations in solution. Among them, the addition of PbBr_2_ gives the most significant chemical shift compared to the pure CrTFA samples without perovskite precursors, which is suggestive of strong coordination between Pb^2+^ and 18Cr6. Similar variations in chemical shifts are also visible in ^19^F NMR with STFA as the reference sample. This owes much to the coordination or ionic bonding between the carboxy unit and the cations (ammonium and/or Pb^2+^) (Fig. 3b). Notably, adding FABr results in the most significant shift of the ^19^F peak, which may arise from the formation of additional hydrogen bonds between ammonium and TFA.

To quantify the binding affinity between the additives and perovskite precursors, we investigated the reaction enthalpy and binding energy by first-principles calculations. We start with the interaction between FABr and STFA, for which the ion exchange process is shown in Eq. (1). The calculated reaction enthalpy is −0.535 eV, indicating that FA^+^ binding to TFA is energetically favored. Next, we calculate the binding energies between 18Cr6 and each metal halide. Our calculation yields a value of −2.795 eV for PbBr_2_ and −2.745 eV for NaBr, indicating both of them can form supramolecular structure with 18Cr6. The small difference in binding energy indicates that NaBr is competitive with PbBr_2_ in their interactions with 18Cr6. The reaction enthalpy for replacing PbBr_2_ with NaBr is as small as 0.286 eV (reaction 2), suggesting that PbBr_2_ could be released during annealing. These results clearly suggest an enhanced driving force for perovskite formation due to the introduction of additional chemical processes.

1 $${{{\mathrm{FABr}}}} + {{{\mathrm{STFA}}}} \to {{{\mathrm{FATFA}}}} + {{{\mathrm{NaBr}}}}$$

2 $$\left\{ {18{{{\mathrm{Cr}}}}6\_{{{\mathrm{PbBr}}}}_2} \right\} + {{{\mathrm{NaBr}}}}\mathop{\longrightarrow}\limits^{\Delta }\left\{ {18{{{\mathrm{Cr}}}}6\_{{{\mathrm{NaBr}}}}} \right\} + {{{\mathrm{PbBr}}}}_2$$

Here, $\left\{ {18{{{\mathrm{Cr}}}}6\_{{{\mathrm{PbBr}}}}_2} \right\}$ represents there is a binding between 18Cr6 and PbBr_2_.

Having revealed the chemical interactions between additives and perovskite precursors, we proceed to study their impact on the resulting films. We first measure the X-ray photoemission spectra (XPS). We show the core level spectra of Pb 4f in Fig. 3d. No noticeable change compared to control films is observable for films with STFA alone. In comparison, we see a slight shift of the Pb 4f peak to higher binding energy for the films with 18Cr6. A striking shift of binding energy is found in the CrTFA films, indicating a significant change in the chemical environment of Pb^2+^.

### Structure analysis by photophysical studies

**Fig. 4:**
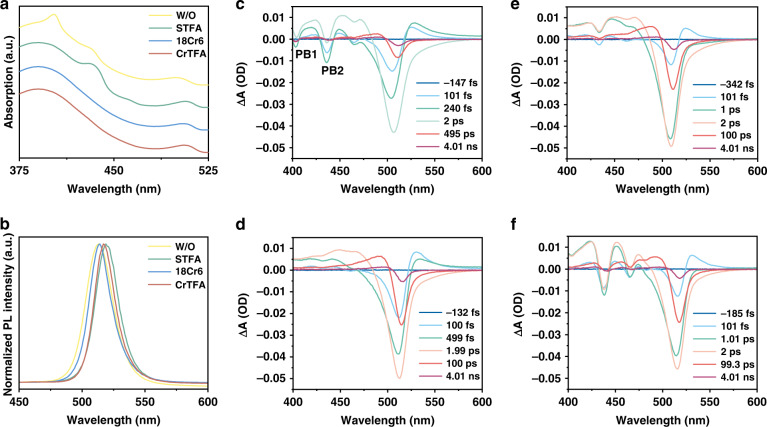
Optical properties of thin films. **a** Absorption and **b** Photoluminescence of thin films. The absorption and emission peaks are slightly red shifted upon addition of additives. Transient absorption spectra of the **c** control films, **d** CrTFA films, **e** 18Cr6 films, and **f** STFA films. The peaks from small layer number are much reduced for the CrTFA films

To gather more information about film composition and thus the rationale for the changes in the chemical environment of Pb^2+^, we studied the photophysical properties of the films by steady-state and transient absorption measurements. The absorption spectra of the control and STFA films show distinct excitonic features peaking at ~403 nm for the former and ~435 nm for the latter (Fig. 4a). Consistently, the photobleaching is situated at close wavelengths (PB1, PB2), which can be assigned to the single-layer and two-layer phases respectively (Fig. 4c, f)^34,35^. In comparison, films with crown as the additive show no sign of exciton features in steady-state absorption, as well as much weaker photobleaching bands in the short wavelength regions (Fig. 4d, e). This suggests that films formed using crown ether are mainly composed of high-dimensional phases with *n* close to infinity, strikingly different from the others. As a result, we observe slightly red-shifted PL emission and narrower PL linewidth with a full width half maximum (FWHM) of ~20 nm compared to that of control and STFA samples (~23 nm) (Fig. 4b).

We proceed to analyze the carrier transfer kinetics based on the TA measurements (Supplementary Fig. 7). In all cases, with the evolution of time, the photobleaching from the low-dimensional phases decreases while the photobleach associated with high-dimensional phases increases, indicating the occurrence of charge transfer^36,37^. Notably, the photobleaching signals assigned to low-dimensional structures attenuate within 1 ns in CrTFA films, which contrasts sharply to the cases where 18Cr6 is not used. The longer carrier lifetime and inefficient charge transfer of control and the STFA films indicate a strong localized separation of perovskite phases that influence carrier transfer.

Having understood the recombination dynamics of our perovskite films, we proceed to investigate their luminescent properties (Supplementary Fig. 8). Among all the cases, CrTFA films show the highest PLQY (~89%) and longest PL lifetime (~82.0 ns), both are larger than the values for 18Cr6 films (60% and 52.7 ns). In comparison, the PLQYs of the control and STFA films are as low as ~10% and PL lifetimes are short (4.5 and 28.7 ns), indicating strong non-radiative recombination. This can be ascribed to the high defect density due to the low crystallinity, as well as the inefficient carrier transfer from low-dimensional to high-dimensional structures^38^.

### Film growth kinetics analysis

**Fig. 5:**
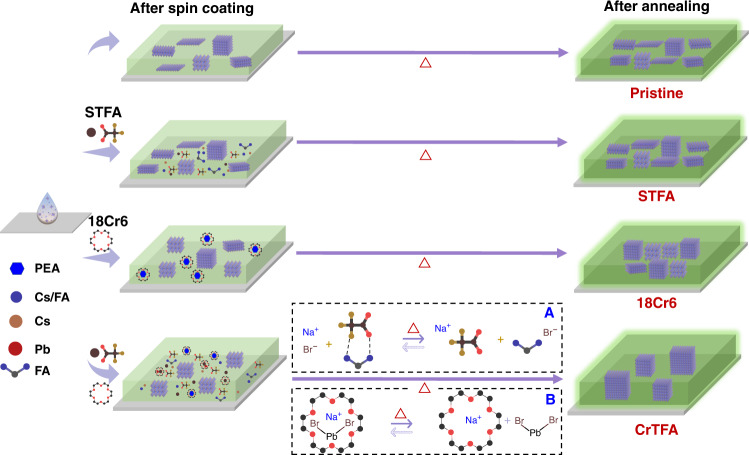
Crystal growth kinetics of films without and with STFA, 18Cr6, and CrTFA. The pristine film with additive shows a large ratio of low-dimensional structure. STFA binding with FABr (reaction A) and 18Cr6 binding with PbBr_2_ suppress the growth of perovskite at room temperature. The annealing process releases FABr and PbBr_2_, giving rise to the second step of crystal growth and large grain size

Overall, based on all characterizations and theoretical simulations above, we are able to show the whole picture of the perovskite crystallization process and its impact on local structures and quality of the films (Fig. 5). 18Cr6 and TFA can strongly bind with lead cations or FA^+^ through coordination or ionic bonding (reaction A and B) (Fig. 5). This results in phase separation of precursors during grain growth and hence a retarded and segmented release of cations for perovskite formation. As a result, a much smaller density of perovskite nanocrystals is formed initially during spin-coating compared to films formed without additive or with a single additive. In the annealing process, NaBr can replace PbBr_2_ to bind with 18Cr6 (reaction B) and hydrogen bonds between TFA and FA are broken (reaction A), enabling the release of ingredients for the second step of growth. The perovskite grains become much larger because of the retarded crystallization and fewer nucleation centers. Together with the low precursor concentration, the formation of nano-island morphology is thus rationalized. Moreover, the interaction between 18Cr6 and PEABr ligands retard the formation of low-dimensional structure leading to a more 3D-like structure, while the large molecules in the surrounding can function as a ligand to passivate the nanocrystal surface. The enhanced crystallinity and surface passivation, the elimination of the 2D phase (*n* = 1), narrowed distribution of 3D-like low-dimensional phases collectively lead to enhanced photoluminescence properties.

### Device performance

**Fig. 6:**
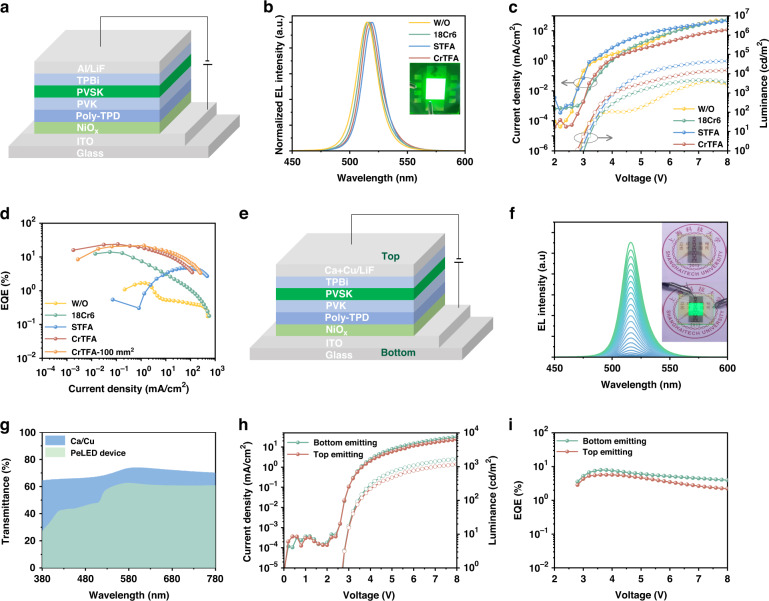
Device performance. **a** Device structure, **b** EL Spectrum, **c** current density–luminance-luminescence (J-V-L), and **d** EQE of devices. **e** Transparent device structure of ITO (180 nm)/NiO_x_ (15 nm)/poly-TPD (15 nm)/PVK (15 nm)/perovskite light emitting layer/TPBi (40 nm)/LiF (1.2 nm)/Ca (8 nm)/Cu (4 nm). **f** Electroluminescence spectra of PeLED from 0 to 8 V. Inset shows a photo of a 100 mm^2^ PeLED. **g** Transmittance of transparent electrodes and whole device. **h** J-V-L of top emitting and bottom emitting of PeLED. **i** External quantum efficiencies of transparent PeLED

We fabricated electroluminescent devices with the architecture of ITO/NiO_x_/Poly-TPD/PVK/Perovskites/TPBi/LiF/Al. Poly-TPD and PVK layers are subsequently deposited onto NiO_x_ (Fig. 6a) to construct a cascaded energy level alignment, which facilitates hole injection^39–41^. The CrTFA devices show a narrow EL linewidth of ~20 nm, consistent with that of PL (Fig. 6b, Supplementary Fig. 9). In all cases, the current density and luminescence increase sharply when the voltage reaches the threshold value. The CrTFA device demonstrates the smallest turn-on voltage, indicating a low series resistance and efficient carrier injection^29^. The maximum luminescence of the CrTFA device is ~14,900 cd m^−2^ at 8 V. The peak external quantum efficiency (EQE) of 23.9% is achieved at a luminescence of 98.8 cd m^−2^ (Fig. 6c). The optimized CrTFA devices with an area of 1 cm^2^ show a peak value of up to 21.6%, which is among the highest for large-area pixels, indicating excellent film homogeneity (Supplementary Table 1)^42^. In comparison, small-area control, STFA, and 18Cr6 display much lower EQE values of 1.7%, 4.7%, and 14.0%, respectively (Fig. 6d), resulting from strong non-radiative recombination and slow carrier transfer.

The high transmittance of the nano-island film provides an excellent opportunity to achieve semi-transparent PeLED, which could be very promising for next-generation smart displays such as luminescent windows (Supplementary Fig. 10). We use a multilayered Ca (8 nm)/Cu (4 nm) as the top electrode (Fig. 6e). The devices show flat transmittance profiles across the visible region from 380 to 780 nm with an average transmittance of 70% and the device shows average transmittance over 50%, which meets the need of trichromatic display applications (Fig. 6g). The EL spectrum and image of transparent PeLED in daylight is shown in Fig. 6f, in which the icon below the device can be clearly observed. Figure 6h shows the current density–luminance-luminescence (J-V-L) of the transparent device. The representative device turns on at a low voltage of ~2.8 V, indicating efficient carrier injection from both electrodes. The emission collected from the ITO side gives a peak of EQE of 7.9%, while the other side provides highest peak value of 5.7% (Fig. 6i). The overall EQE is up to 13.6%, the highest value for a PeLED with transmittance over 50%.

## Discussion

In summary, we have developed a supramolecular strategy to regulate the crystallization kinetics of green emissive perovskites by using dual additives, which can selectively interact with both A-site and B-site cations in perovskite. The supramolecular structures retard crystal growth and reduces the number of nucleation centers, which eventually bring narrowed distribution of low-dimensional phases and a nanocrystal structure. The film shows excellent luminescent quantum yield and fast carrier injection, leading to a light emitting diode with external quantum yields of 23.9%. The segmented crystallization brings excellent film homogeneity, giving rise to an efficiency of 21.6% for a 1 cm^2^ large-area device. In addition, a semi-transparent device with an efficiency of up to 13.6% was developed. All these results are among the best realized to date. This work clarifies the working mechanism of additives and demonstrated an effective strategy for the growth of high-quality perovskite emitter, and thus sheds light on growing high-quality perovskite films for large-area and transparent displays.

## Methods

### Materials

Dimethyl sulfoxide (DMSO; 99.9%), 18-Crown-6 (18Cr6), hydrobromic acid (HBr), Poly (9-vinylcarbazole) (PVK), lithium fluoride (LiF), cesium bromide (CsBr), chlorobenzene, and xylene were purchased from Sigma-Aldrich. PbBr_2_ and sodium trifluoroacetate (STFA) was purchased from TCI. Ethanolamine and Nickel (II) acetate tetrahydrate was purchased from Alfa Aesar. Phenethylamine (PEA) was purchased from Adamas. Poly[N,N’-bis (4-butylphenyl)-N,N’-bisphenylbenzidine] (poly-TPD) from Xi'an p-OLED company. Formamidinium bromide (FABr; ≥99.99%) from Greatcell Solar Materials. 1,3,5-Tris (1-phenyl-1H-benzimidazol-2-yl) benzene (TPBi) from Jilin OLED material tech.

### Synthesis of PEABr

20 mmol of phenethylamine was dissolved in 20 mL of ethanol and vigorously stirred, and 40 mmol of HBr was added dropwise to the flask. Subsequently, the mixture is steamed at 60 degrees. Finally, the precipitate is cleaned with ether and dried in a vacuum oven.

### Perovskite precursor solution

The control precursor solution was prepared by mixing PbBr_2_, CsBr, FABr, and PEABr with a molar ratio of 1:0.85:0.15:0.4 in DMSO (PbBr_2_ is 0.2 M). The precursor was stirred at 37 °C for 12 h. For CrTFA precursor, 3.5 mg of 18Cr6 (0.014 mmol) and STFA (0.026 mmol) were added to the precursor.

### NiO_x_ precursor solution

The preparation of precursor solution was conducted by a reported method^43^. Equimolar quantities of Nickel (II) acetate tetrahydrate and ethylenediamine were added to an ethylene glycol solution. Then the solution (0.2 M) was stirred at 60 °C for 4 h.

### LED fabrication and EL characterization

The ITO glasses were cleaned with Triton X-100, isopropanol, and deionized water with ultrasonic cleaning for 30 min each. Then the glasses were exposed under O_2_ plasma for a quarter of an hour. After that, NiO_x_ precursor was spin-coated the substrates and baked under 300 °C for 45 min covered with a big glass dish. Then, poly-TPD (6 mg mL^−1^ in chlorobenzene) layers and PVK (4 mg mL^−1^ in xylene) was spin-coated onto the NiO_x_ at 3000 rpm and annealed on a hot plate for 20 min at 130 °C in a glovebox. The precursor was spin-coated onto the PVK substrate at 3000 rpm for 1 min, and annealed at 80 °C for 5 min. Last, 40 nm of TPBi, 1.2 nm of LiF, and Al (120 nm) electrodes were deposited under a based vacuum of ~7 × 10^−7^ torr.

The current density, luminance, and EQE values were tested by a Keithley 2612B and an integrating sphere (FOIS-1) with a QE Pro650 spectrometer (SpectrumTEQ-EL system). The EQE of LED can be defined as ${{{\mathrm{EQE}}}} = \frac{{{\rm{emitted}}\;{\rm{photons}}\;{\rm{out}}\;{\rm{LED/second}}}}{{{\rm{injected}}\;{\rm{electrons/second}}}}$. The counts of photons per second are collected by an integrating sphere and a fiber spectrometer and the counts of electrons are collected with Keithley 2612B source meter. The LED devices were tested on top of the integrating sphere, and only forward light emission could be collected, which is consistent with the standard OLED characterization method. All the device test processes were performed in the N_2_-filled glovebox. The EL data of the 100 mm^2^ devices were measured by PR-655 spectrometer with a Keithley 2400.

### Film characterization

Cary 5000 (Agilent) was used to measure absorption of perovskite thin films. PL, PLQY, and time-resolved photoluminescence (TRPL) was measured by Flog-3 (HORIBA). TA was measured using a HELIOS femtosecond transient absorption spectrometer (Ultrafast Systems LLC) with a 365-nm laser (5 μW). USB2000 + spectrometer (Ocean Insight) was used to track the in-situ PL spectrum, and the films were excited with a 365 nm LED light soure. Surface and cross section SEM picture was measured by JEOL7800. JEOL-2100Plus was used to characterize the perovskite grains under 200 keV. D8 (Bruker) was used to measure the XRD data with Cu Kα source (λ = 1.5405 Å). To protect the wet film from humidity and solvent atmosphere, the XRD test is performed right after spin-coating and the film is sealed in a larger sealed bag full of nitrogen before test. The measurement chamber was kept in a low humidity with nitrogen atmosphere. Nuclear Magnetic Resonance (NMR) were measured by Avance III HD500 (Bruker). ESCALAB 250Xi (Thermo Fisher) was used to collected the XPS data. FTIR was measured using Bruker VERTEX 70 using ATR mode. AFM-IR was measured using nanoIR2-FS (Anasys).

### First-principles calculations

Density functional theory calculations are performed by the projector-augmented wave method implemented in the Vienna Ab initio Simulation Package (VASP)^44,45^. We employ the generalized gradient approximation parameterized by Perdew, Burke, and Ernzerhof, with the plane-wave cutoff energy of 400 eV. The van der Waals interaction is treated with the DFT-D2 method^46,47^. For binding energy calculation, the molecules are put into a 25 × 25 × 25 Å^3^ periodic unit cell. The convergence criteria for structure relaxation is that the force on each atom is smaller than 0.01 eV Å^−1^.

## Supplementary information

Supporting Information for In-situ growth of low-dimensional perovskite-based insular nanocrystals for highly efficient light emitting diodes
