# Supplementary material for: In-situ growth of low-dimensional perovskite-based insular nanocrystals for highly efficient light emitting diodes
Source: Light Sci Appl. 2023 Mar 3;12:62. doi: 10.1038/s41377-023-01112-7 (PMC9984476; doi:10.1038/s41377-023-01112-7)
Supplement: Supplementary file 1 — Supporting Information for In-situ growth of low-dimensional perovskite-based insular nanocrystals for highly efficient light emitting diodes [file 41377_2023_1112_MOESM1_ESM.docx]

**Supporting Information for**

**In-situ growth of low-dimensional perovskite-based insular nanocrystals for highly efficient light emitting diodes**

Hao Wang^1†^, Weidong Xu^2†^, Qi Wei^1^, Si Peng^1^, Yuequn Shang^1^, Xianyuan Jiang^1^, Danni Yu^1^, Kai Wang^1^, Ruihua Pu^1^, Chenxi Zhao^1^, Zihao Zang^1^, Hansheng Li^1^, Yile Zhang^1^, Ting Pan^1^, Zijian Peng^1^, Xiaoqin Shen^1^, Shengjie Ling^1^, Weimin Liu^1^, Feng Gao^3^* & Zhijun Ning^1^*

^1^School of Physical Science and Technology, ShanghaiTech University, Shanghai, China.

^2^Institute of Flexible Electronics, Northwestern Polytechnical University, China

^3^Department of Physics, Chemistry and Biology (IFM), Linköping University, Linköping, Sweden

^†^ These authors contributed equally to this work.

*Corresponding author: feng.gao@liu.se, ningzhj@shanghaitech.edu.cn

KEYWORDS: Perovskite, Light-emitting diodes, Low-dimensional, Nanocrystal, Transparent devices

**Supplementary Figures**


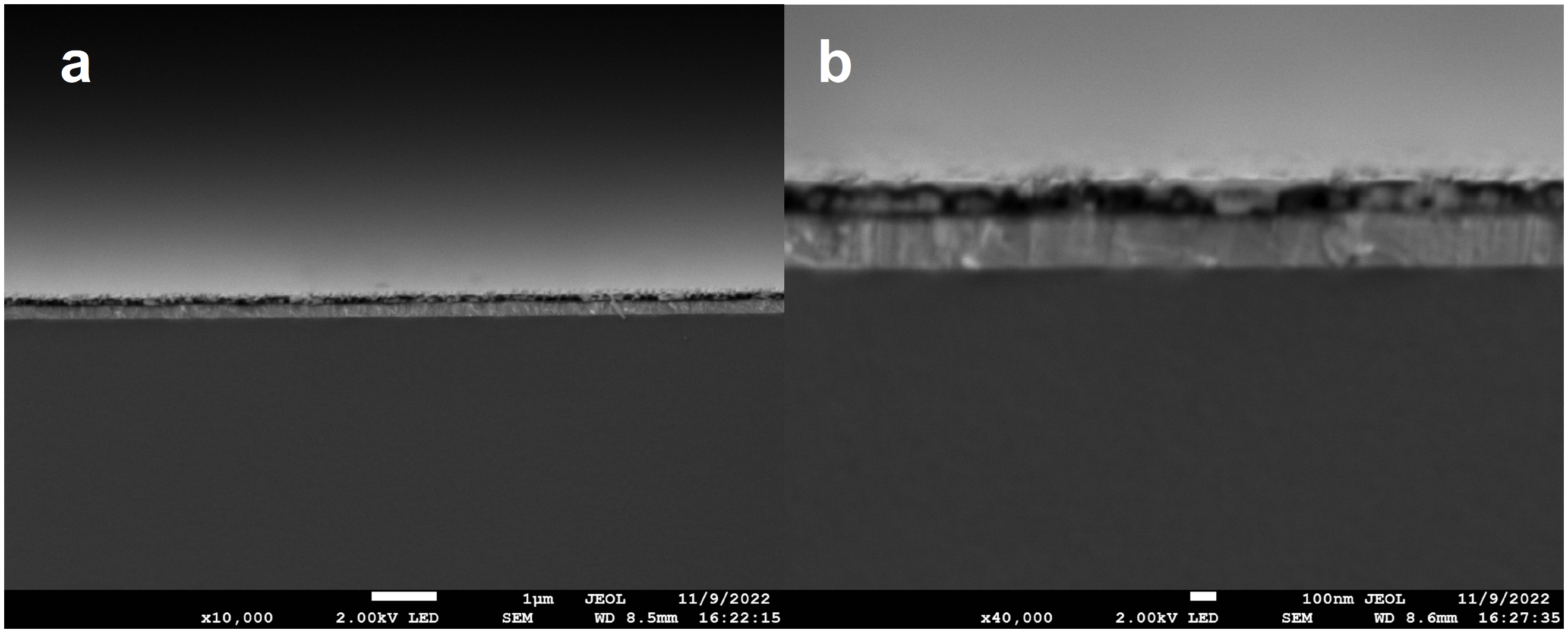


**Supplementary Fig. 1.** The amplified cross section images of CrTFA film shown in Fig. 1d.


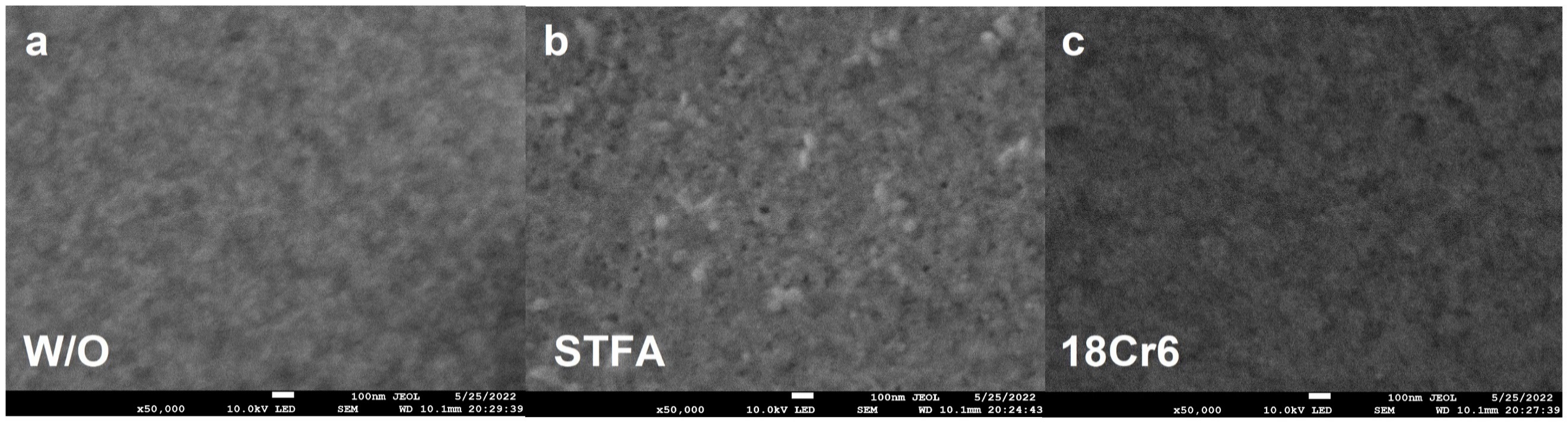


**Supplementary Fig. 2.** Surface SEM images of (a) control, (b) STFA, and (c) 18Cr6 films (Scale bar is 100 nm).


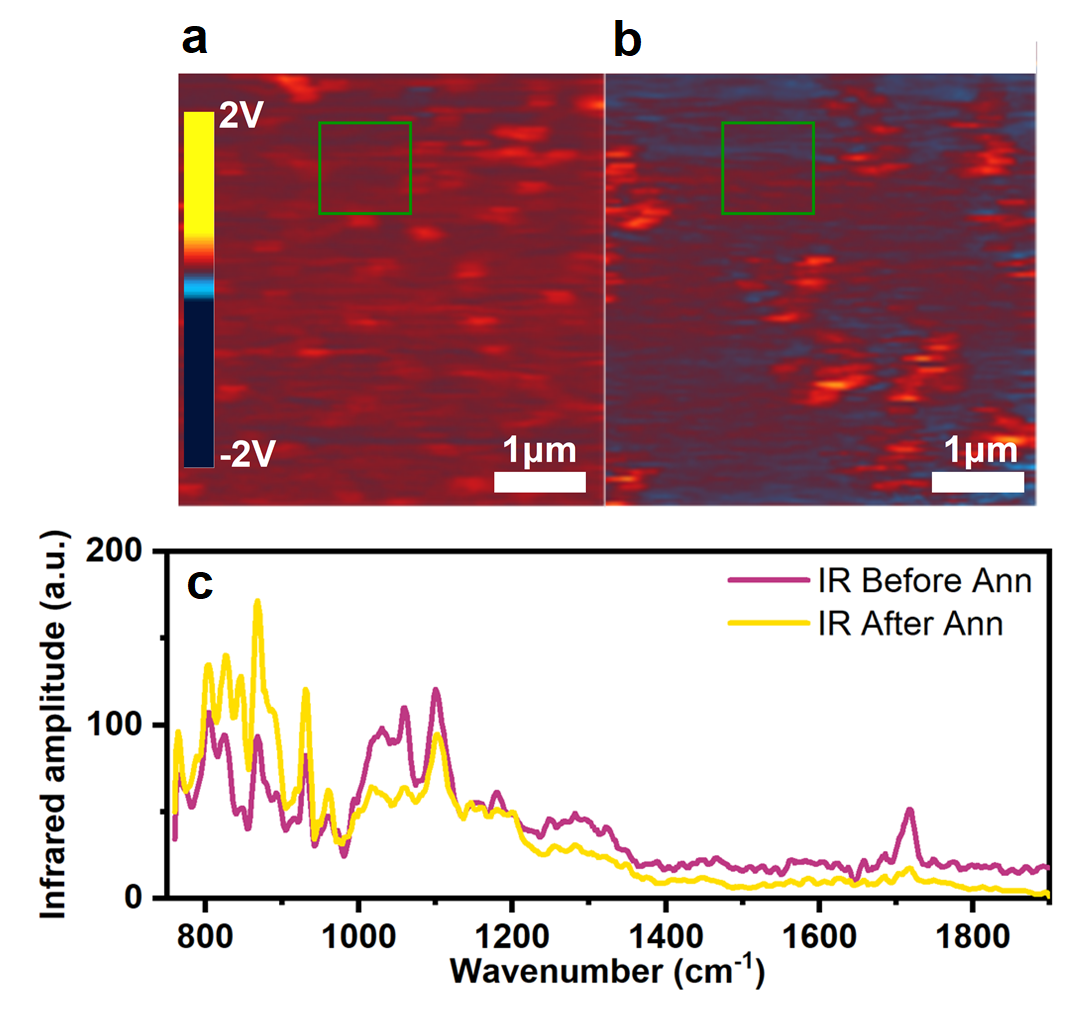


**Supplementary Fig. 3.** AFM-IR (1710 cm^-1^) images of CrTFA film (a) before and (b) after annealing. (c) IR spectra of selected micro areas (green boxes above) in films before and after annealing using AFM-IR. An obvious peak at 1710 cm^-1^ is observed for the film before annealing, and the peak is much weak for the film after annealing.


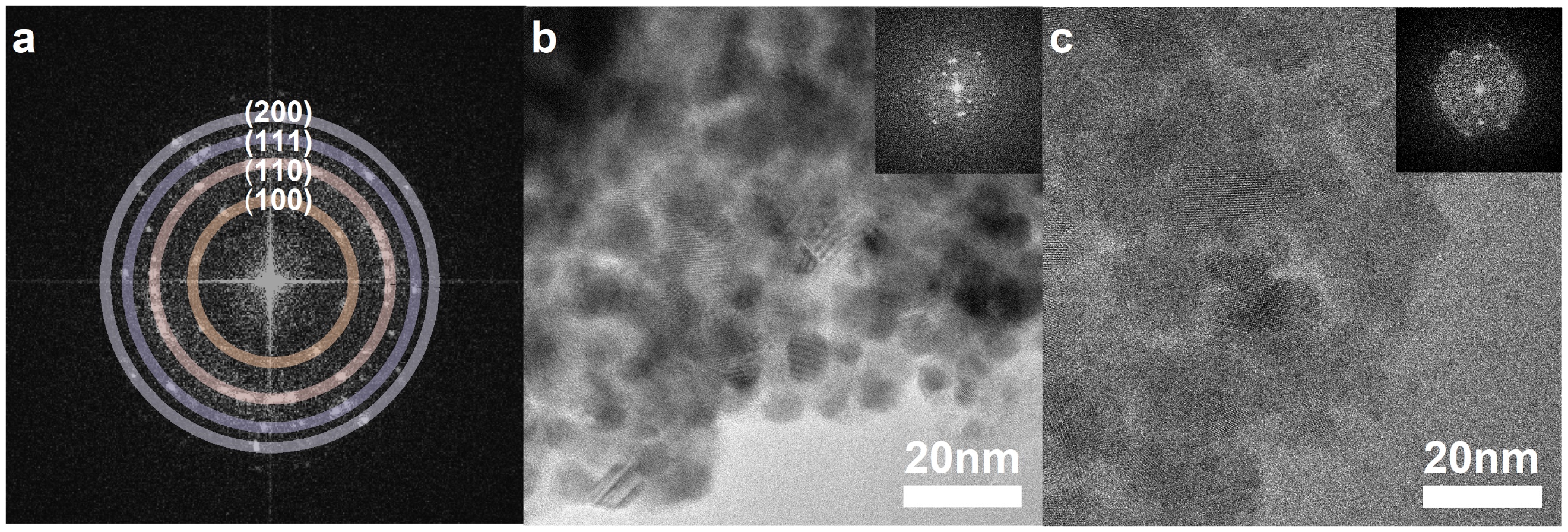


**Supplementary Fig. 4.** (a) FFT of the TEM image of the CrTFA film shown in Fig. 1e, TEM images of (a) 18Cr6 and (c) STFA films.


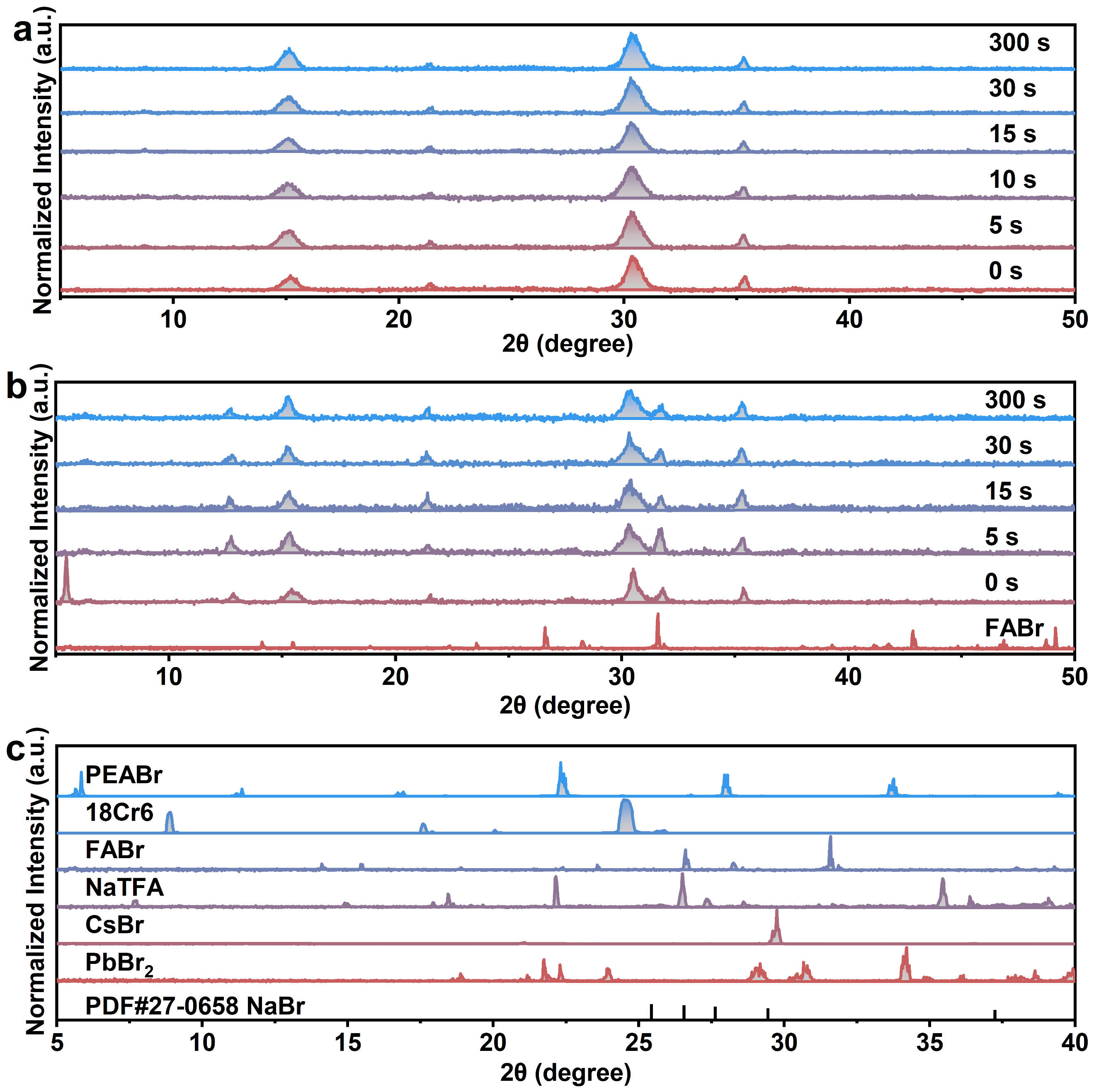


**Supplementary Fig. 5.** XRD spectra evolution of the (a) 18Cr6 film and (b) STFA film during annealing and (c) XRD spectra of each component in perovskite precursor.
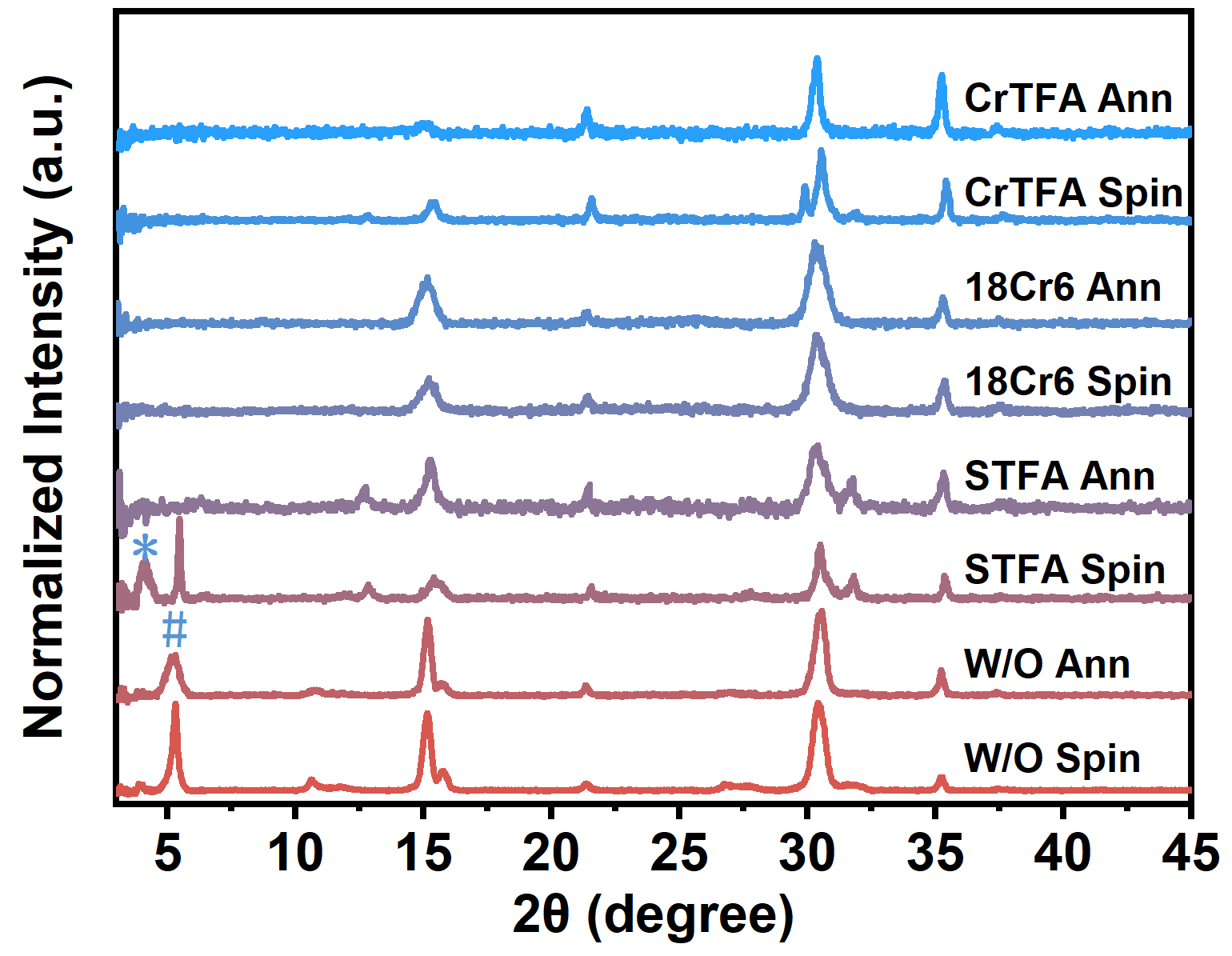


**Supplementary Fig. 6.** XRD spectra of films after spin-coating and annealing (* and # represents n=2 and n=1 layer respectively). The control film and the STFA film show strong peak from 2D structure, while this peak is not observed for the 18Cr6 films.


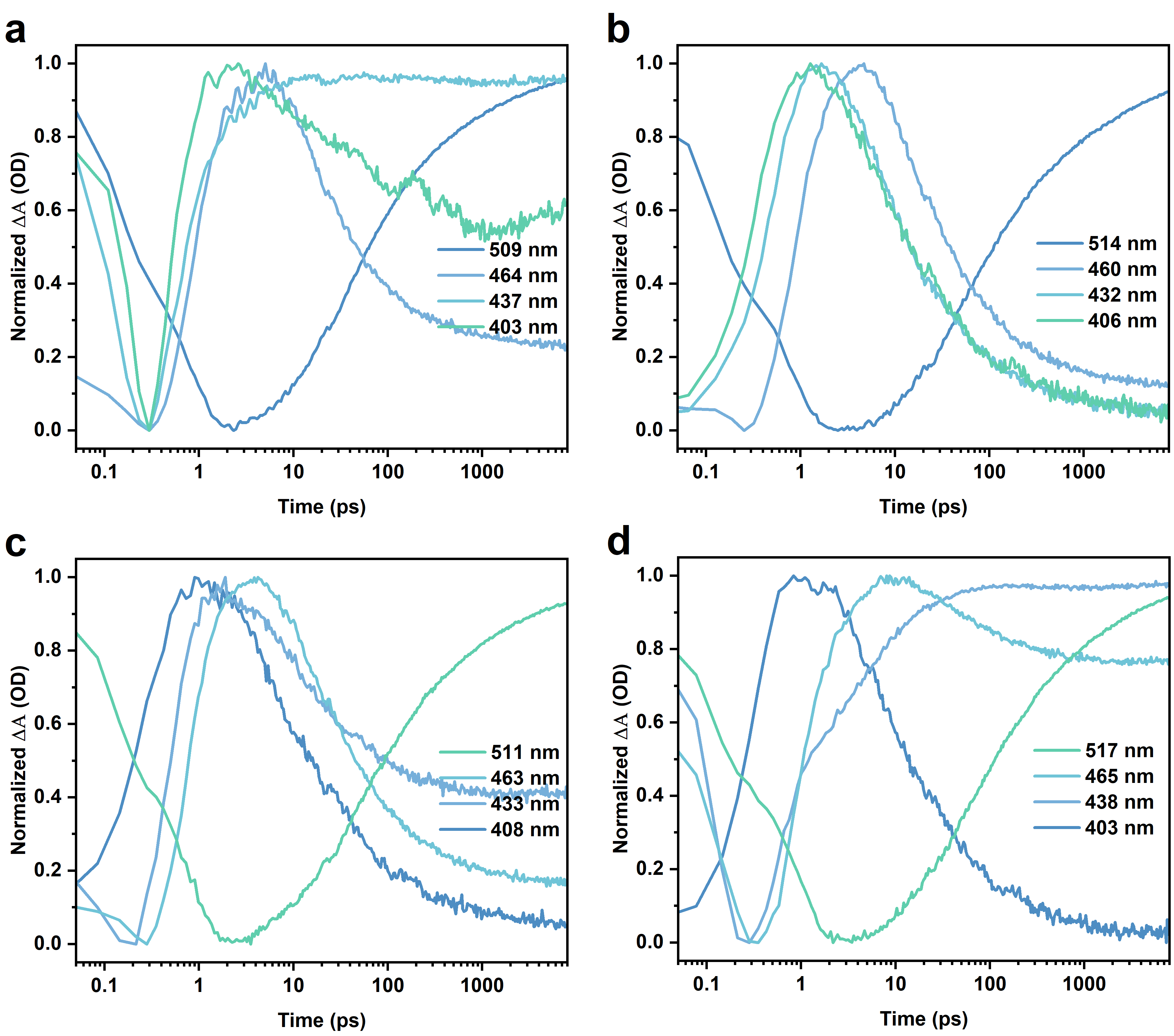
 **Supplementary Fig. 7.** Kinetic of each bleaching peak in transient absorption spectra of thin film of the (a) control, (b) CrTFA, (c) 18Cr6, and (d) STFA.


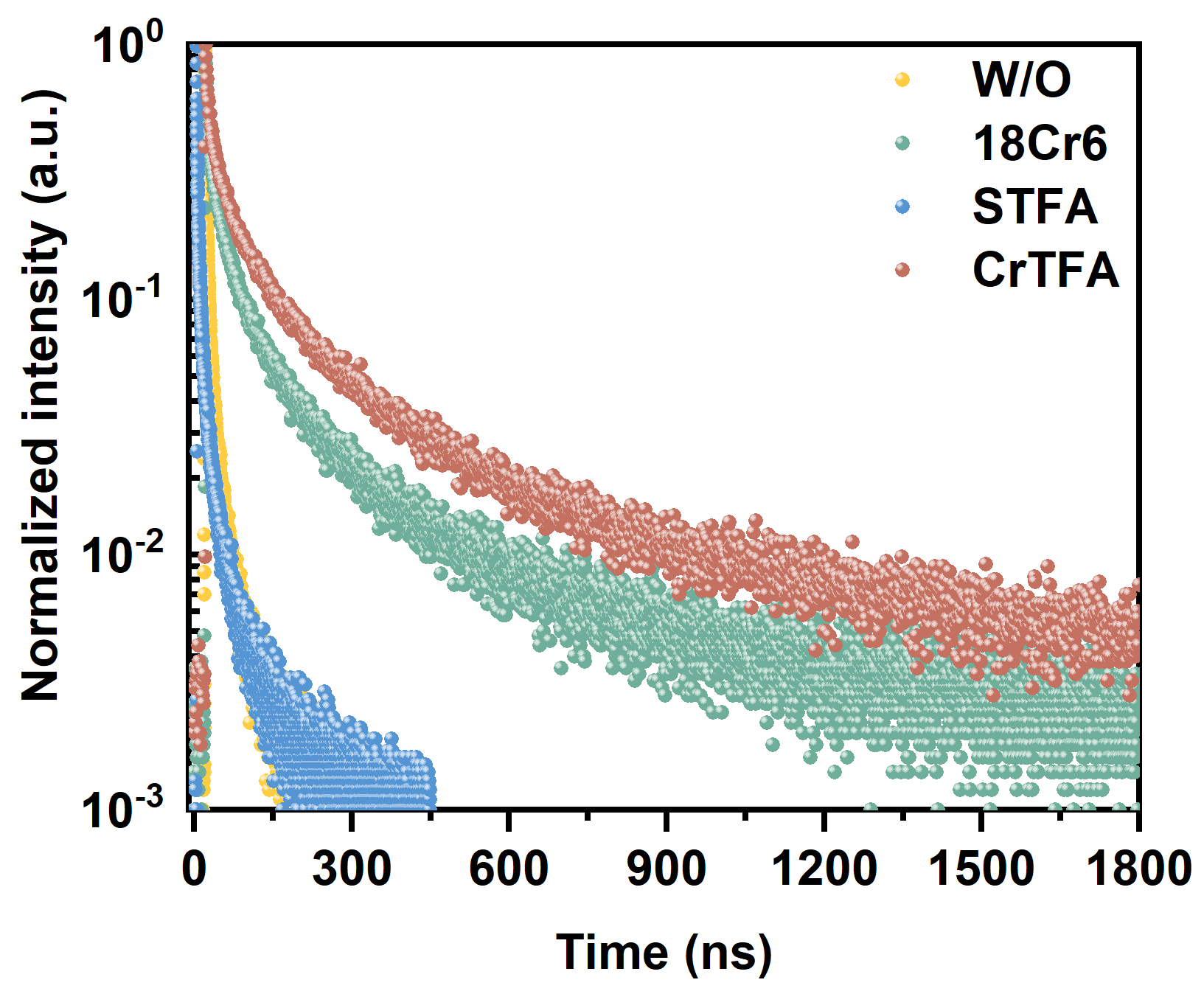


**Supplementary Fig. 8.** TRPL for the control, 18Cr6, STFA, and CrTFA film, with respective lifetime of 4.5, 52.7, 28.7, and 82.0 ns.


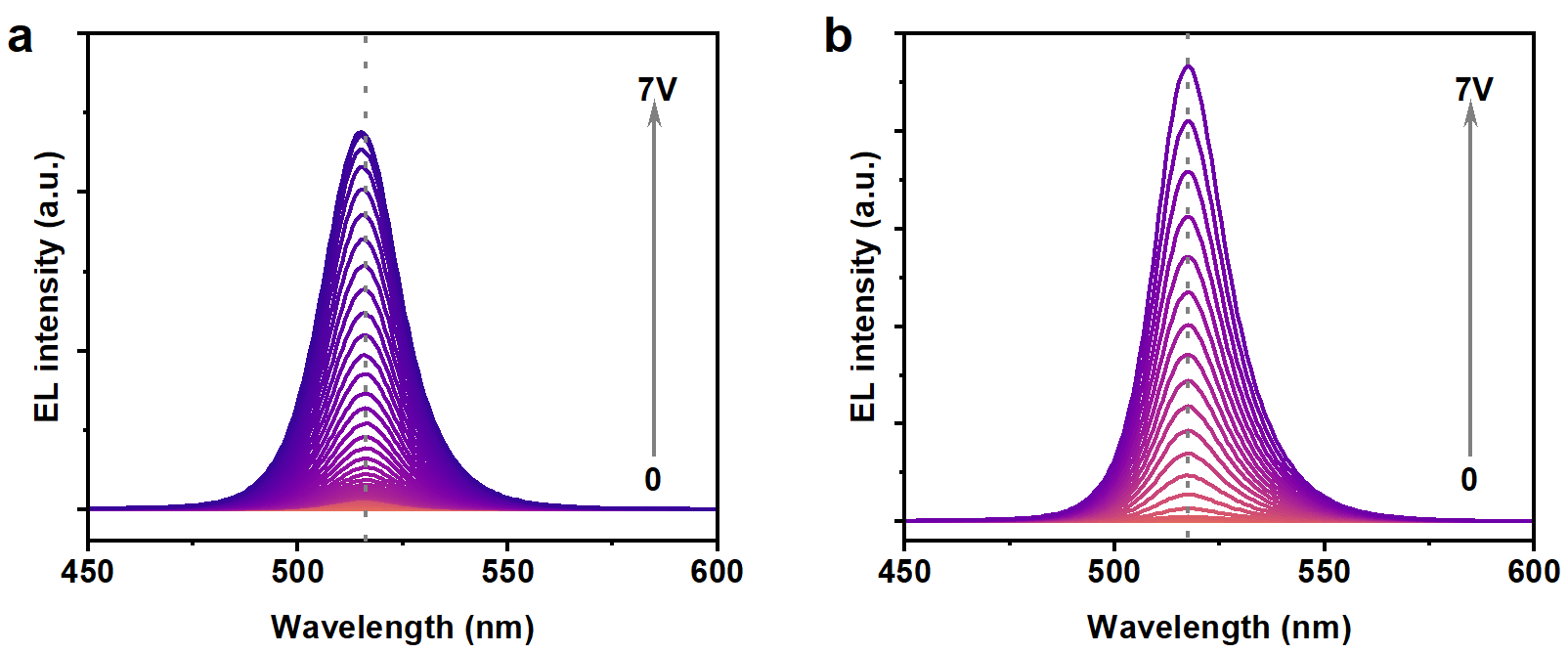


**Supplementary Fig. 9.** EL spectrum of the (a) control and (b) CrTFA film. With the increase of voltage, the EL peak of CrTFA film does not change, but the EL peak of the control film has obvious blue shift.


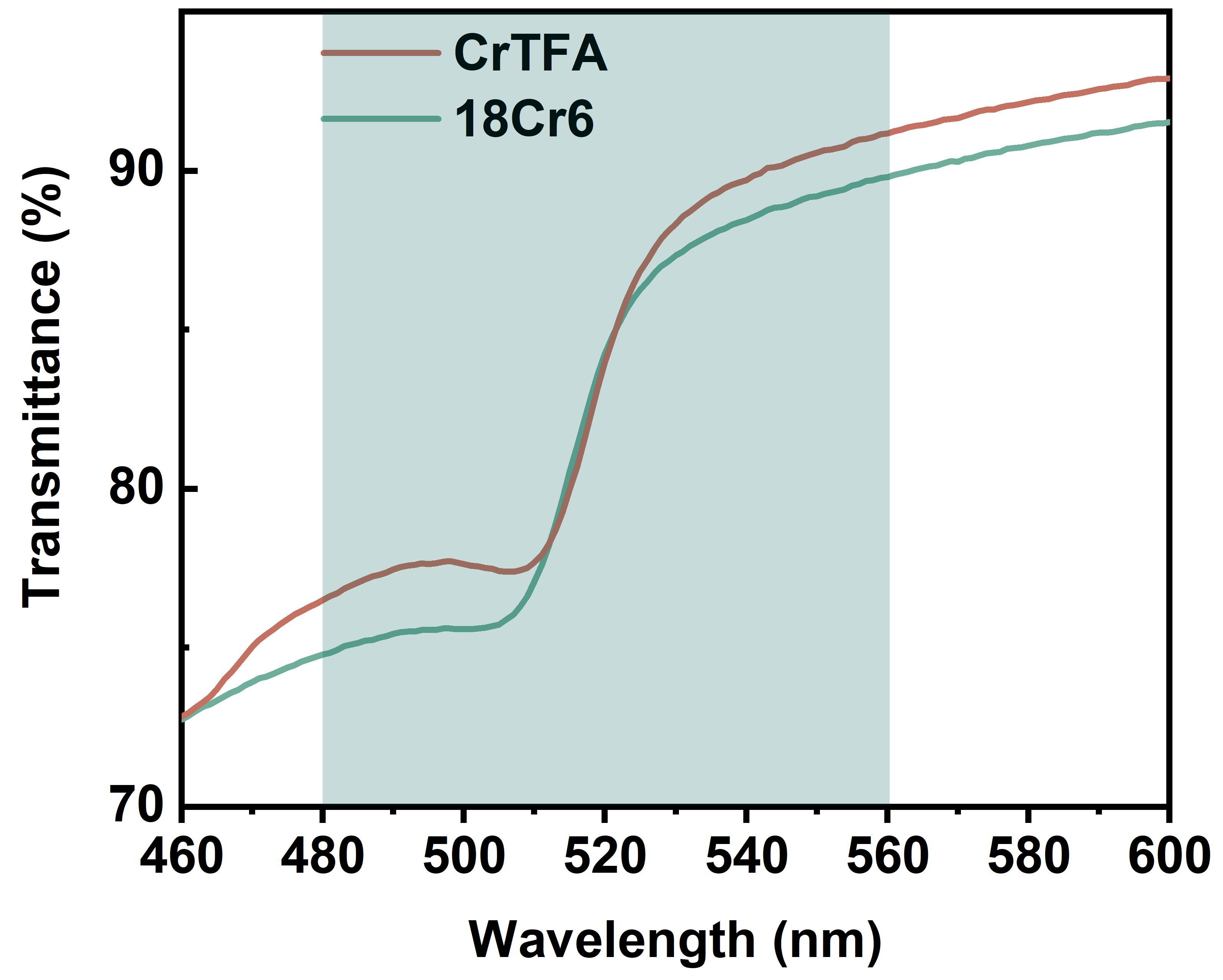


**Supplementary Fig. 10.** The transmittance of different perovskite thin films.


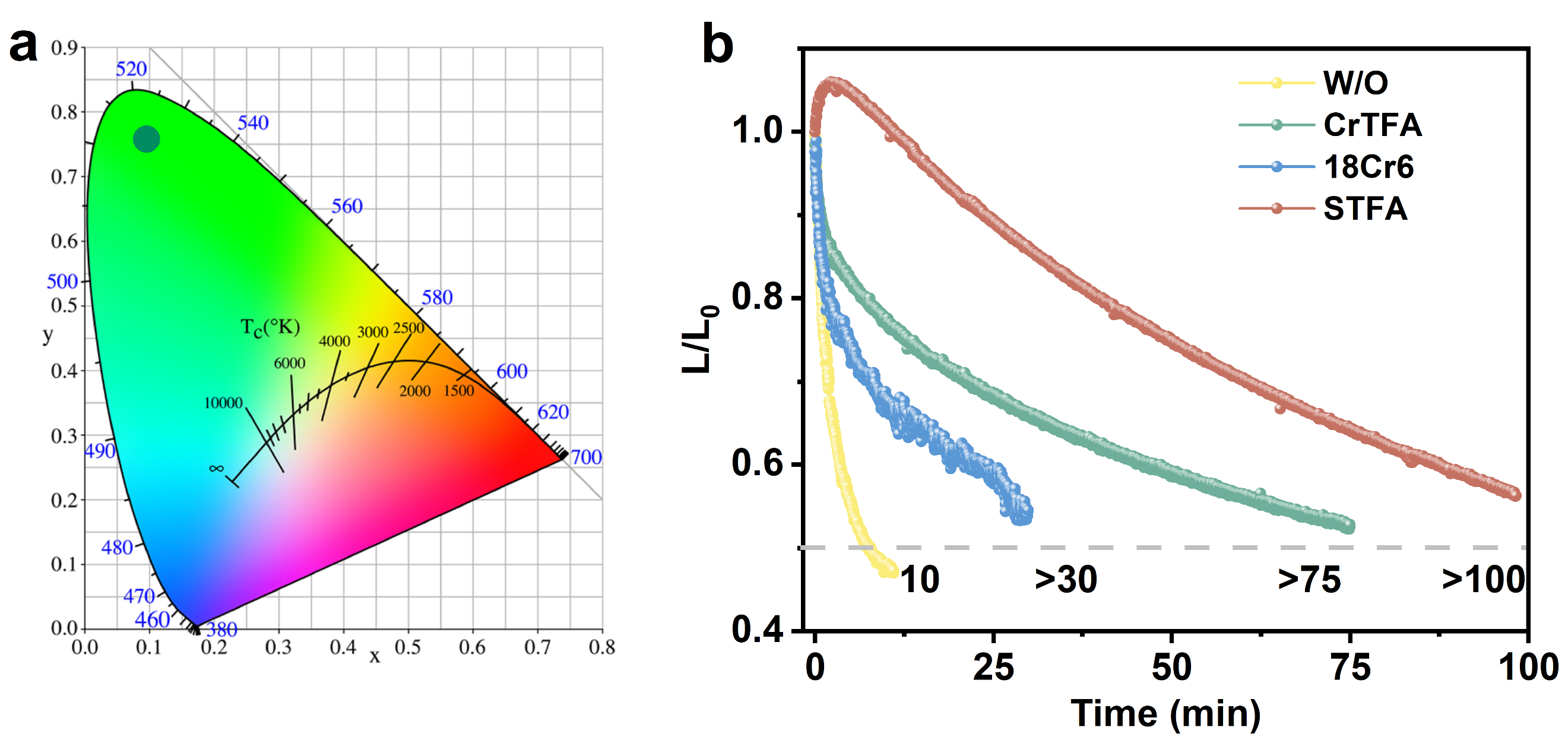


**Supplementary Fig. 11.** (a) CIE chromaticity coordinates (b) EL lifetime of devices with STFA (L_0_=105 cd m^-2^), with 18Cr6 (L_0_=110 cd m^-2^), without (L_0_=113 cd m^-2^) and with CrTFA (L_0_=107 cd m^-2^).

**Supplementary Table 1.** Representative device performance of large active area perovskite green light emitting diode.

| Perovskite | Active area  (cm^2^) | EQE  (%) | λ  (nm) | Methods | Reference |
| --- | --- | --- | --- | --- | --- |
| PEABr: CsPbBr_3_: NVAL | 9 | 16.4 | 520 | Spin – coatting | 1 |
| FAPbBr_3_ NCs | 4 | 16.3 | 531 | Spin – coatting | 2 |
| CsPbBr_3_ | 40.2 | 7.1 | 508 | Thermal evaporation | 3 |
| CsPbBr_3_ NCs | 4 | ~11 | 512 | Spin – coatting | 4 |
| FAPbBr_3_ nanoplatelets | 3 | 2 | 530 | Spin – coatting | 5 |
| PEABr: CsPbBr_3_: CrTFA | 1 | 21.6 | 516 | Spin – coatting | This work |

**Supplementary Table 2.** Representative device performance of small active area perovskite green light emitting diode.

| Perovskite | Active area  (mm^2^) | EQE  (%) | λ  (nm) | Reference |
| --- | --- | --- | --- | --- |
| FA_0.9_GA_0.1_PbBr_3_ PNCs | N/A | 23.4 | 531 | 6 |
| PEABr: CsPbBr_3_ with crown: MPEG-MAA | 10 | 28.1 | 514 | 7 |
| PEA_2_Cs_1.6_MA_0.4_Pb_3_Br_10_: TFPPO | 5.25 | 25.2 | 517 | 8 |
| PEABr: CsPbBr_3_: LiBr | 3.24 | 23.6 | 518 | 9 |
| PEA_2_PbBr_4_(CsPbBr_3_)_4_: ETPTA | 10 | 22.5 | 508 | 10 |
| PEABr: CsPbBr_3_: CrTFA | 20 | 23.9 | 516 | This work |

**Supplementary Table 3.** Representative device performance of transparent light emitting diodes.

| Emitting layer | EQE_Top_ (%) | EQE_Bottom_ (%) | λ  (nm) | Average transmittance (%) | Reference |
| --- | --- | --- | --- | --- | --- |
| CdSe/ZnS QDs | ~4 | ~6 | ~530 | 84  (380 nm – 780 nm) | 11 |
| FAPbI_3_ | 1.2 | 4.5 | 799 | 55  (450 nm – 650 nm) | 12 |
| PEABr: CsPbBr_3_ | 6.5 | 9.6 | 512 | <50  (350 nm – 650 nm) | 13 |
| PEABr: CsPbBr_3_ | 5.7 | 7.9 | 516 | > 50  (380 nm – 780 nm) | This work |

**References**

1 Sun, C. *et al.* High-performance large-area quasi-2D perovskite light-emitting diodes. *Nat. Commun.* **12**, 2207, (2021).

2 Wang, H. *et al.* A Multi-functional Molecular Modifier Enabling Efficient Large-Area Perovskite Light-Emitting Diodes. *Joule* **4**, 1977-1987, (2020).

3 Du, P. *et al.* Efficient and large-area all vacuum-deposited perovskite light-emitting diodes via spatial confinement. *Nat. Commun.* **12**, 4751, (2021).

4 Yuan, S. *et al.* Self-Assembled High Quality CsPbBr_3_ Quantum Dot Films toward Highly Efficient Light-Emitting Diodes. *ACS Nano* **12**, 9541-9548, (2018).

5 Kumar, S. *et al.* Ultrapure Green Light-Emitting Diodes Using Two-Dimensional Formamidinium Perovskites: Achieving Recommendation 2020 Color Coordinates. *Nano Lett.* **17**, 5277-5284, (2017).

6 Kim, Y.-H. *et al.* Comprehensive defect suppression in perovskite nanocrystals for high-efficiency light-emitting diodes. *Nat. Photonics* **15**, 148-155, (2021).

7 Liu, Z. *et al.* Perovskite Light-Emitting Diodes with EQE Exceeding 28% through a Synergetic Dual-Additive Strategy for Defect Passivation and Nanostructure Regulation. *Adv. Mater.* **33**, 2103268, (2021).

8 Ma, D. *et al.* Distribution control enables efficient reduced-dimensional perovskite LEDs. *Nature* **599**, 594-598, (2021).

9 Cui, J. *et al.* Efficient light-emitting diodes based on oriented perovskite nanoplatelets. *Sci. Adv.* **7**, eabg8458, (2021).

10 Chu, Z. *et al.* Perovskite Light-Emitting Diodes with External Quantum Efficiency Exceeding 22% via Small-Molecule Passivation. *Adv. Mater.* **33**, 2007169, (2021).

11 Choi, M. K. *et al.* Extremely Vivid, Highly Transparent, and Ultrathin Quantum Dot Light-Emitting Diodes. *Adv. Mater.* **30**, 1703279, (2018).

12 Xie, C., Zhao, X., Ong, E. W. Y. & Tan, Z.-K. Transparent near-infrared perovskite light-emitting diodes. *Nat. Commun.* **11**, 4213, (2020).

13 Cai, L. *et al.* High-Efficiency Top-Emitting Green Perovskite Light Emitting Diode with Quasi Lambertian Emission. *Adv. Opt. Mater.* **10**, 2101137, (2022).
